# Comparative study on dynamic visual tracking abilities in three-dimensional multi-object tracking tasks among basketball players of different skill levels

**DOI:** 10.3389/fpsyg.2025.1710450

**Published:** 2025-11-05

**Authors:** Zhi Guo, Qiulin Wang

**Affiliations:** College of Physical Education, Yangzhou University, Yangzhou, Jiangsu, China

**Keywords:** three-dimensional multi-object tracking, basketball players, dynamic visual processing, attention features, tracking ability

## Abstract

**Objective:**

This study aimed to examine whether high-level basketball players exhibit superior multi-object tracking abilities compared to low-level basketball players using the three-dimensional multi-object tracking (3D-MOT) task paradigm.

**Methods:**

Forty-eight participants (24 high-level and 24 low-level basketball players) from a university in Jiangsu were recruited. A 2 × 4 mixed experimental design was employed, with group (high vs. low) and tracking load as independent variables, and reaction time and accuracy in the 3D-MOT task as dependent variables.

**Results:**

The main effect of tracking load was significant, with tracking accuracy showing a linear decrease as the number of targets increased. The main effect of athletic level was also significant: while both groups showed reduced performance with increasing target numbers, high-level basketball players maintained higher accuracy and shorter reaction times under greater tracking loads.

**Conclusion:**

High-level basketball players outperformed low-level basketball players in multi-object tracking tasks, demonstrating superior adaptability and stability in dynamic visual information processing. Specifically, they maintained more accurate and efficient tracking performance when faced with complex task demands.

## 1 Introduction

Dynamic and unpredictable sport environments place high demands on athletes’ attentional capacities (Guo and Wang, 2025). Basketball, for example, is a fast-paced, highly interactive team sport in which players must monitor multiple moving targets simultaneously, including teammates, opponents, the ball, and other contextual factors. During play, athletes are required not only to focus on ball handling and passing but also to continuously perceive and evaluate the spatial positions and trajectories of multiple dynamic elements (Liang et al., 2005). Such demands align closely with research on multi-object tracking tasks (MOT), which emphasizes the ability to sustain attention to several moving targets in order to guide timely and appropriate responses (Meyerhoff et al., 2017).

To investigate this ability Pylyshyn and Storm (1988), proposed the classic MOT paradigm, which evaluates dynamic attentional allocation and resource management through three stages: target presentation, tracking, and response (Meyerhoff et al., 2017). Previous studies have shown that athletes outperform non athletes in MOT tasks, particularly when the number of targets increases or when target speed is high (Pylyshyn and Storm, 1988). For example Qiu et al. (2018), found that elite college basketball athletes performed better than non-athletes when tracking three or more targets, and Li (2012) reported that expert college basketball players maintained higher accuracy than novices as target number increased. Similarly Jin et al. (2020), demonstrated that athletes outperformed college students when target speed increased. Comparisons across sports also indicate that athletes possess advantages in visual information processing speed, with basketball players showing especially strong performance in MOT tasks, particularly guards when target number increases (Li, 2012). Importantly, MOT ability has been linked to actual competitive performance. In professional basketball, players with stronger MOT ability record more assists and steals and commit fewer turnovers during a season (Mangine et al., 2014). These findings suggest that MOT is not only a laboratory-based cognitive task but also a predictor of athletic performance.

However, most prior studies have relied on traditional two-dimensional (2D) MOT tasks, which lack sufficient ecological validity. In real environments, depth information in three-dimensional space is critical for judging and estimating object motion. Monocular and binocular depth cues enhance depth perception and thereby improve dynamic target tracking (Loyola, 2018). Compared with 2D tasks, three-dimensional MOT (3D-MOT) more accurately reflects the complexity of real sport environments and allows comprehensive assessment of spatial attention allocation, visual search efficiency, and dynamic decision making (Taian et al., 2019). The 3D-MOT paradigm has been widely used in cognitive psychology and sports science research to measure dynamic attention and visuospatial processing. Previous studies have demonstrated its reliability, with high test–retest stability and consistent performance patterns across sessions (Deschamps et al., 2022; Zhang et al., 2025). Evidence indicates that 3D-MOT not only increases target discriminability and tracking accuracy but also reveals superior performance in professional athletes compared to amateurs and no athletes, with training effects transferring to actual competition (Faubert, 2013; Guoy et al., 2024). These findings suggest that 3D-MOT provides greater ecological validity and practical value for both sport training and cognitive research.

Although numerous studies have examined athlete performance in MOT tasks, systematic comparisons across different levels of basketball players remain limited. In particular, little is known about basketball players’ multiple object tracking abilities and potential cognitive advantages in 3D-MOT contexts. Therefore, the present study employed a 3D-MOT paradigm to compare players of different skill levels on dynamic attentional allocation and tracking performance. The findings are expected to enrich theoretical understanding in sport cognition and to provide practical implications for training, performance enhancement, visual–cognitive interventions, and physical education strategies. By revealing cognitive advantages of highly skilled basketball players in MOT, this study aims to inform the design of individualized training programs and support improvements in athletes’ decision-making and response speed under complex competitive conditions.

Hypotheses: (1) High-level basketball players are expected to demonstrate greater tracking accuracy than low-level players as the task load increases in the 3D-MOT paradigm. (2) High-level basketball players will exhibit superior dynamic attentional allocation, reflected in faster tracking speed compared with low-level players.

## 2 Research methods

### 2.1 Participants

This study focused on the dynamic visual tracking abilities of basketball players, following the visual search paradigms commonly used in sports psychology research (Guoy et al., 2024). Because the present study employed a two-way repeated-measures ANOVA, an *a priori* power analysis was conducted using G*Power (version 3.1). Based on meta-analytic findings from prior research (Guoy et al., 2024), the expected effect size was set to a medium magnitude (*f* = 0.30), the probability of a Type I error was set at *α* = 0.05, and the desired statistical power (1 − *β*) at 0.95. The analysis indicated that a minimum of 36 participants would be required. To account for an anticipated attrition rate of approximately 20% and to further ensure the robustness of the findings, we recruited a total of 48 participants for the present study. We choose 48 male basketball players from different universities in Jiangsu, China, participated in the study. Participants were divided into two groups based on athletic skill level: 24 high-level players and 24 low-level players. The high-level group consisted of athletes who were certified as at least national first-level players, each with experience competing in the CUBAL first league. The low-level group consisted of athletes certified as national second-level players, who had received systematic basketball training and regularly participated in university-level competitions, but had not competed at the higher national first-league level. On average, the high-level group reported longer training histories than the low-level group. All participants were right-handed, had normal or corrected-to-normal vision, and were physically and mentally healthy, with no intellectual or psychological impairments. All provided written informed consent and received compensation upon completion of the experiment. The experiment was approved by the Ethics Committee of the Medical School of Yangzhou University (YXYLL-2025-129). Detailed demographic and background information for the participants are presented in Table 1.

**Table 1:** Information of study participants.

Group	Number of participants	Athletic level	Training experience (years)	Age
High-level athletes	24	National first-class athletes	9.04 ± 1.06	20.42 ± 1.25
Low-level athletes	24	National second-class athlete	3.96 ± 1.17	19.95 ± 1.5

### 2.2 Experimental design

Previous studies have shown that factors such as the number, speed, and shape of the tracked targets can be used to manipulate tracking load, with the number of targets being widely recognized as the most influential factor (Guoy et al., 2024). Therefore, this study increased tracking load by varying the number of targets. A 2 (Skill Level: High, Low) × 4 (Tracking Load: 3–6) mixed experimental design was adopted, and a 3D-MOT task was used to assess the participants’ tracking performance.

### 2.3 Apparatus and materials

The experiment was conducted on a Lenovo Think Book 16 laptop equipped with a 16-inch display (1,920 × 1,080 resolution; 60 Hz refresh rate). The 3D-MOT task was programmed in MATLAB R2024a.

### 2.4 Experimental protocol

The 3D-MOT paradigm was implemented in the present study because it offers a reliable and valid measure of dynamic visual tracking performance. Prior research has confirmed its test–retest reliability and construct validity in sports contexts (Deschamps et al., 2022; Meyerhoff et al., 2017). Moreover, manipulating the number of targets is a well-established procedure for adjusting task load, ensuring both methodological rigor and comparability with existing findings (Marion et al., 2025).

**Figure 1:**
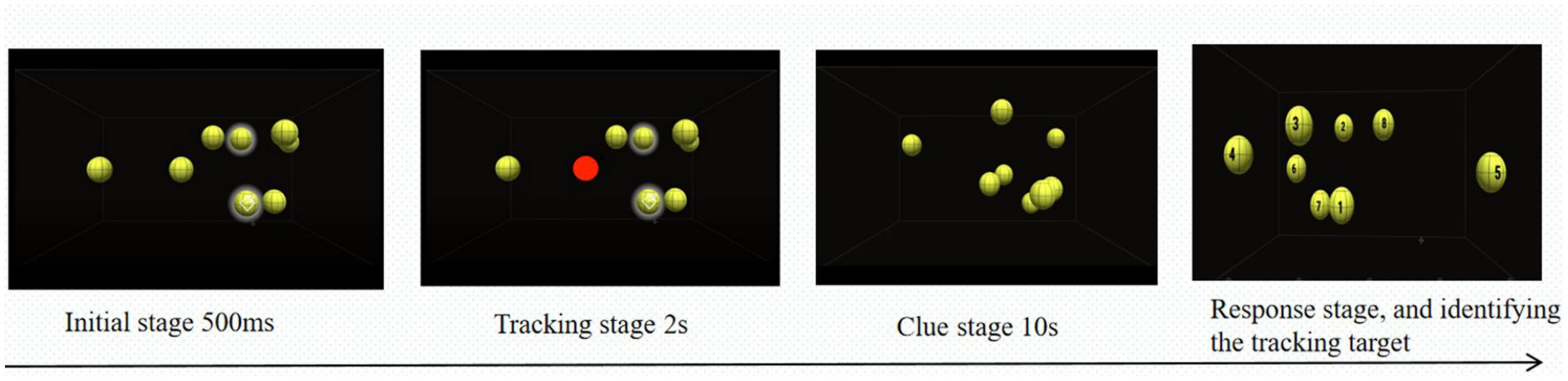
Flowchart of the 3D-MOT task process.

At the beginning of each trial, 10 orange-yellow balls were presented against a black background. Among them, 3–6 balls briefly changed color from orange to red and blinked three times (2 s per blink), designating them as target balls, while the remaining balls served as distractors. Subsequently, all balls reverted to their original color and moved randomly within the space at a constant speed of 5°s. After 10 s, the balls stopped moving and were labeled with numbers from 0 to 9. Participants were instructed to identify the target balls by pressing the corresponding numeric keys before proceeding to the next trial. The experiment consisted of four blocks, each corresponding to a different number of targets, with 30 trials per block (see Figure 1).

The between-group independent variable was skill level (high vs. low), and the within-group independent variable was tracking load (number of targets: 3–6). The dependent variables were tracking accuracy and response time. Accuracy was calculated as the percentage of trials in which participants correctly selected all the target balls, while response time was defined as the average time taken by participants from the beginning of the tracking response phase until all target balls were labeled (Michaels et al., 2022).

### 2.5 Experimental procedure

Each participant was brought individually into the laboratory. First, the participant was asked whether they felt any physical discomfort. A folder was created using the participant’s name and ID to save and export experimental data. The experimental equipment and room lighting were adjusted. The purpose of the experiment and important precautions during the procedure were then introduced to the participant. The participant was instructed to sit with their head fixed, eyes focused on the display, and positioned 60 cm from the screen. Once the head position was adjusted and fixed, the experimental instructions were displayed. The participant underwent a nine-point calibration. Five practice trials were provided, during which the participant familiarized themselves with the experimental operations and key selections. After completing the practice trials, the formal experiment began.

### 2.6 Statistical analysis

All data were analyzed using SPSS 26.0 (IBM Corp., Armonk, NY, USA). Descriptive statistics were calculated and data were checked for normality and homogeneity of variance prior to inferential analyses. A 2 (group: high-level vs. low-level) × 4 (tracking load: 2–5 targets) mixed-design analysis of variance (ANOVA) was conducted, with group as the between-subjects factor and tracking load as the within-subjects factor. When significant main or interaction effects were observed, *post hoc* pairwise comparisons with Bonferroni correction were performed to control for Type I error. In addition, effect sizes were calculated to evaluate the magnitude of differences, with partial eta squared (*η^2^ₚ*) reported for ANOVA effects. The level of statistical significance was set at *p* < 0.05.

## 3 Results

### 3.1 Accuracy analysis of 3D multiple-object tracking across groups

**Figure 2:**
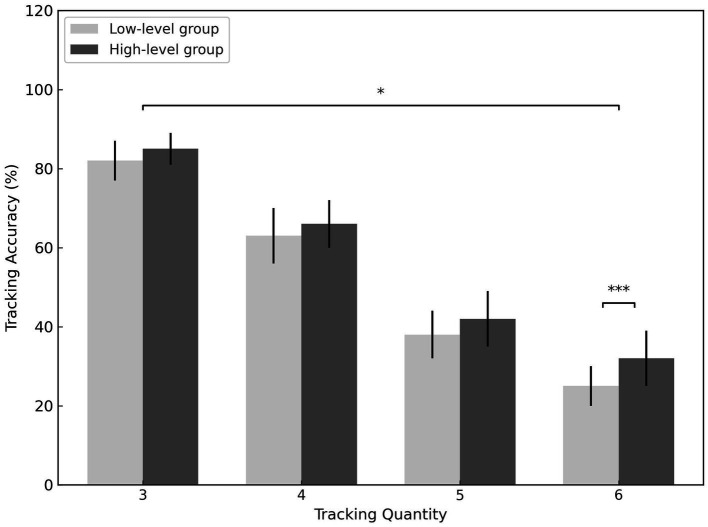
A comparative study on tracking accuracy between high-level basketball athletes and low-level athletes. ^***^p < 0.001, ^*^p < 0.05.

The results indicated a significant main effect of skill level (*F* = 12.409, *p* = 0.001, *η^2^ₚ* = 0.212), showing that high-level athletes performed significantly better than low-level athletes in tracking accuracy. A significant main effect of tracking load was also found (*F* = 766.313, *p* < 0.001, *η^2^ₚ* = 0.980), with accuracy rates significantly decreasing as the number of targets increased for both groups. Furthermore, a significant interaction between skill level and tracking load was observed (*F* = 3.028, *p* = 0.032, *η^2^ₚ* = 0.184), indicating that the interaction of these two variables influenced tracking accuracy (see Figure 2).

Further simple effects analysis revealed that when tracking 3, 4, or 5 targets, there was no significant difference in accuracy between high-level and low-level athletes. However, when the number of targets increased to 6, the accuracy rate of high-level athletes was significantly higher than that of low-level basketball players (*p* < 0.001, *η^2^ₚ* = 0.304).

### 3.2 Reaction time analysis of 3D multiple-object tracking across groups

**Figure 3:**
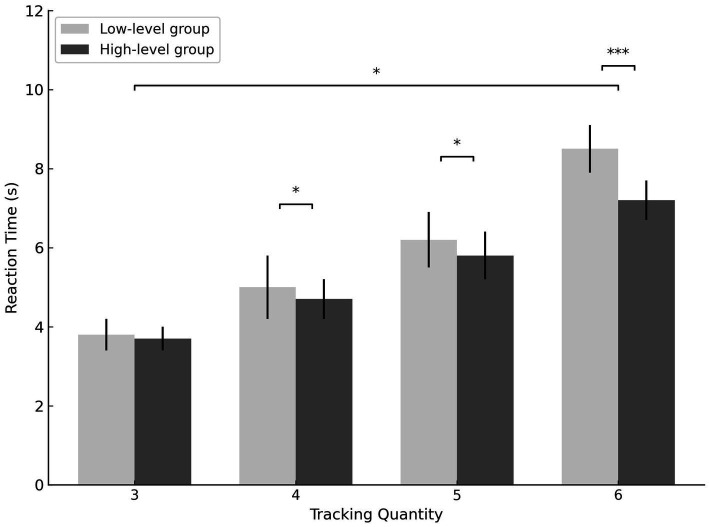
A comparative study on tracking reaction time between high-level basketball athletes and low-level athletes. ^***^p < 0.001, ^*^p < 0.05.

The results indicated a significant main effect of skill level, with high-level athletes exhibiting significantly faster reaction times than low-level athletes (*F* = 14.841, *p* < 0.001, *η^2^ₚ* = 0.244). A significant main effect of tracking load was also observed (*F* = 313.475, *p* < 0.001, *η^2^ₚ* = 0.872), with reaction times significantly increasing as the number of targets increased for both groups. Moreover, a significant interaction between skill level and tracking load was found (*F* = 5.548, *p* = 0.004, *η^2^ₚ* = 0.108), indicating that the interaction of these two variables influenced tracking reaction times (see Figure 3).

Further simple effects analysis revealed no significant difference in reaction times between high-level and low-level athletes when tracking 3 targets. However, when tracking 4 targets, high-level athletes exhibited significantly faster reaction times than low-level athletes (*p* = 0.047, *η^2^ₚ* = 0.083). Similarly, when tracking 5 targets, high-level athletes showed significantly faster reaction times compared to low-level athletes (*p* = 0.025, *η^2^ₚ* = 0.105). When the number of targets increased to 6, high-level athletes still demonstrated significantly faster reaction times than low-level athletes (*p* < 0.001, *η^2^ₚ* = 0.232).

## 4 Discussion

The present findings indicate that high-skilled basketball players performed significantly better than low-skilled players on the 3D-MOT task, demonstrating higher tracking accuracy, faster response times, and greater performance stability. These results support the view that sports training may enhance higher-order cognitive functions and highlight the potential positive effects of basketball-specific practice on spatial attention and dynamic perception (Faubert, 2013; Liao and Zhang, 2006).

### 4.1 Influence of skill level on tracking performance

The present study demonstrated that high-skilled basketball players outperformed low-skilled players on the 3D-MOT task, exhibiting higher tracking accuracy, faster response times, and greater performance stability. This finding is consistent with previous research suggesting a positive association between sport skill level and MOT ability (Guoy et al., 2024). It is noteworthy that high-level athletes usually have longer training histories than lower-level athletes, which is consistent with the natural progression of skill development—athletes who reach higher competitive levels generally accumulate more years of training experience (Baker et al., 2003; Menting et al., 2019). Therefore, the superior performance of high-level athletes compared with low-level athletes in this study may reflect not only differences in competitive level but also the advantages conferred by extended training experience.

Such advantages are likely attributable to long-term systematic training and extensive competitive experience. Through years of practice, basketball players develop stable knowledge structures and strategic modules related to their sport, encompassing technical–tactical skills, cognitive processing, and psychological regulation. When confronted with complex multi-target tracking situations, these structures allow players to rapidly activate relevant knowledge, efficiently extract, integrate, and update information, and thus achieve superior performance (Guo and Wang, 2025). This capacity renders high-skilled basketball players closer to an “ideal observer,” able to use motion direction, spatial position, and feature cues to establish target correspondence while maintaining target representations through visual working memory and attentional switching. However, some studies have reported no significant association between sport skill level and MOT performance (Jin et al., 2020; Scharfen and Memmert, 2019). Such discrepancies may be due to differences in experimental paradigms and task demands. The present study employed a 3D-MOT paradigm, which is closer to real-world scenarios and incorporates depth cues that may better reveal athletes’ advantages. In addition, when task load is low, group differences may be masked. For instance Li (2012), found no significant difference between experts and novices when tracking fewer than five objects, suggesting that five targets may represent a threshold for distinguishing performance levels. Consistent with this view, the current results showed that high-skilled basketball players exhibited a significant advantage only under the six-target condition. Nevertheless, the effect sizes of these group differences were relatively small, indicating that while statistically reliable, the magnitude of the advantage should be interpreted with caution in terms of its practical significance in real-game contexts.

Further research has suggested that differences in MOT performance may also depend on sport type Harris et al. (2020) reported that team sport athletes (e.g., soccer, rugby) performed better than individual sport athletes (e.g., track and field, swimming), whereas Memmert et al. (2009) found no such differences, possibly due to variations in task design, such as self-selected movement speeds. Overall, most evidence supports a positive relationship between sport expertise and MOT performance, and this advantage may reflect the transfer of domain-specific expertise to general cognitive domains (Alves et al., 2013). Such transfer is thought to occur when training and transfer tasks share overlapping cognitive processes (Dahlin et al., 2008). In particular, athletes in open-skill team sports are required to monitor multiple dynamic targets in constantly changing environments and to allocate limited attentional resources efficiently (Guo and Wang, 2025).

In conclusion, the present findings provide further evidence of cognitive advantages in high-skilled basketball players with respect to MOT and dynamic attentional allocation. These results contribute to understanding the relationship between motor expertise and cognition and have practical implications for basketball training. Specifically, 3D-MOT–based training may serve as an effective approach to enhance visual tracking and decision-making, help athletes adapt to complex game environments, and offer a useful tool for player selection and performance evaluation.

### 4.2 Influence of tracking load on performance

The present study showed that tracking performance declined in both groups of basketball players as the number of tracked targets increased, indicating that task load gradually heightened. This finding is consistent with previous research (Guoy et al., 2024) and reflects the limited capacity of attentional resources. According to the fixed-slot theory (Scholl et al., 2001; Yi et al., 2004), attentional capacity is typically 4 ± 1 items; as the number of targets increases, fewer resources can be allocated to each target, thereby reducing accuracy. The perceptual load hypothesis (Lavie, 2006) further suggests that when participants cannot track all objects, they selectively allocate attention to certain targets while treating distractors as background to reduce resource demands.

Importantly, although accuracy and response time declined with increasing task load in both groups, high-skilled players showed a smaller decrement, suggesting that they can maintain performance more effectively under high cognitive load. This advantage is likely related to the demands of basketball competition. During transitions, fast breaks, and defensive rotations, athletes must track multiple rapidly moving objects within very short time frames and allocate limited attention to critical targets (Peng et al., 2020). High-skilled players appear more capable of sustaining attention on the ball while concurrently monitoring teammates’ positions and opponents’ movements, thereby maintaining greater stability and efficiency under high-load conditions (Lozzi et al., 2025). Furthermore, the present study employed a 3D-MOT paradigm, which incorporates depth information beyond that available in 2D tasks. Monocular cues (e.g., relative size, occlusion, texture gradients, shading) and binocular disparity provide richer spatial information that enhances target discriminability (Li and Pizlo, 2011). Consistent with this Cooke et al. (2017), reported higher tracking accuracy in 3D than 2D MOT, particularly when target spacing increased, enabling faster tracking performance. The present findings align with this evidence, further indicating that depth cues facilitate attentional allocation under high-load conditions. Nonetheless, the relatively modest effect sizes suggest that both groups may have approached their cognitive processing limits, which reduces the observable magnitude of between-group differences. Consequently, future studies should examine whether similar patterns hold under more ecologically valid and game-like conditions.

In summary, high-skilled basketball players demonstrated superior performance in multiple-target, high-load conditions, reflecting cognitive advantages in attentional resource allocation and dynamic task processing. These results extend theoretical understanding of load effects on attention and provide practical implications for basketball training. The superior tracking performance observed in high-level players suggests that dynamic visual tracking may be a trainable skill that develops with systematic practice and competitive experience. Coaches may therefore consider incorporating 3D-MOT–based drills or other dynamic attention training exercises into regular practice to strengthen athletes’ ability to monitor multiple moving objects simultaneously. For novice and lower-level players, such training may accelerate the development of attentional allocation strategies, improve their ability to anticipate opponents’ movements, and enhance situational awareness during games. For higher-level players, training with progressively increased tracking loads may help maintain attentional sharpness under the demanding conditions of fast-paced competition. Integrating perceptual–cognitive training with basketball-specific drills could thus provide a complementary approach to traditional skill and physical training, ultimately enhancing decision-making and performance in real-game contexts.

## 5 Limitations and future research directions

This study has several limitations. First, the sample size was modest, consisting of 48 male basketball players recruited from several universities in Jiangsu Province. The exclusion of female athletes and the limited geographical scope restrict the generalizability of the findings. In addition, the study did not include a novice or non-athlete group, which limits the ability to fully compare tracking performance across different levels of expertise. Moreover, individual differences such as playing position were not considered, even though previous studies have suggested that guards may demonstrate particular advantages in dynamic visual tracking. Future research should therefore aim to expand the participant pool by including female athletes, recruiting from a wider range of regions, incorporating novice participants, and examining the role of playing position to provide a more comprehensive understanding of expertise-related differences in dynamic visual tracking. Second, although participants were divided by competitive level, the groups were not matched on training years. This reflects the natural progression of skill development, as athletes who attain higher certification levels generally accumulate more years of training experience, but it also makes it difficult to fully disentangle the effects of competitive level and training history. Third, although statistically significant differences were observed, some effect sizes were relatively small, suggesting that the practical implications of the findings should be interpreted with caution. Fourth, the experiment was conducted in a controlled laboratory environment, which lacks the ecological complexity, situational pressure, and physical demands of real basketball games, thereby limiting ecological validity. Fifth, individual differences in attentional capacity and cognitive strategies were not controlled for, and these may have influenced task performance within groups. Finally, although previous studies have reported acceptable reliability and validity for the 3D-MOT paradigm, its psychometric properties in sport-specific contexts remain less established. Future research should therefore employ larger and more diverse samples, consider longitudinal designs to separate skill level from training history, adopt more ecologically valid paradigms, and further examine the task’s measurement properties in relation to real-game performance.

## 6 Conclusion

High-level basketball players outperform low-level basketball players in MOT, demonstrating superior adaptability and advantages in dynamic visual information processing. Specifically, they maintain higher tracking accuracy and faster tracking speed, especially when faced with complex tasks.

## Data Availability

The original contributions presented in the study are included in the article/supplementary material, further inquiries can be directed to the corresponding author.
